# Identification of the pericardiacophrenic vein on CT

**DOI:** 10.1186/s40644-017-0134-4

**Published:** 2018-01-05

**Authors:** Yoshiyuki Ozawa, Ritsuko Suzuki, Masaki Hara, Yuta Shibamoto

**Affiliations:** 1 Department of Radiology, Nagoya City University, Graduate School of Medical Sciences, 1 Kawasumi, Mizuho-cho, Mizuho-ku, Nagoya, 467-8601 Japan; 2 Department of Radiology, Nagoya City West Medical Center, Nagoya, Japan

**Keywords:** Anterior mediastinum, Tumor, Phrenic nerve, Ct, Thymoma

## Abstract

**Background:**

To evaluate the depictability of pericardiacophrenic veins (PCPV) as landmarks for the location of the phrenic nerves on multi-detector-row computed tomography (MDCT), and to investigate the usefulness of depicting the PCPV to aid differential diagnosis of anterior mediastinal lesions.

**Methods:**

Fifty-six patients with anterior mediastinal lesions (Fifty lesions originated from the thymus, six were of non-thymic origin) were evaluated. Contrast-enhanced CT scans of the chest were performed in all cases before diagnosis, and 22 of these scans were performed with electrocardiographic (ECG) gating. Two chest radiologists assessed the depictability of the PCPV and the positional relationship between the center of each anterior mediastinal lesion and the ipsilateral PCPV.

**Results:**

The use of ECG gating increased the PCPV depiction rate in the lower left part of the mediastinum. The depiction rate of the left PCPV was significantly higher than that of the right PCPV. All 50 tumors of thymic origin and 3 of the 6 tumors of non-thymic origin were located on the medial side of the ipsilateral PCPV. The 3 lesions located on the lateral side of the ipsilateral PCPV were of non-thymic origin (*p* = 0.0007).

**Conclusion:**

The use of ECG gating during MDCT may improve the depictability of the PCPV in the lower left section of the anterior mediastinum. Solitary anterior mediastinal lesions located on the lateral side of the ipsilateral PCPV are likely to be of non-thymic origin.

## Background

The phrenic nerves are small and difficult to detect on computed tomography (CT), and no previous studies have examined the positional relationship between anterior mediastinal tumors and the phrenic nerves, or the clinical significance of identifying the phrenic nerves in such cases.

Anterior mediastinal tumors, such as thymic epithelial tumors, mediastinal lymphomas, and germ cell tumors, mainly arise from the thymus [1]. However, some non-thymic lesions also develop in the anterior mediastinum, and it is sometimes difficult to diagnose these anterior mediastinal lesions based on morphological information alone. To identify additional sources of information that can facilitate the diagnosis of such tumors, we hypothesized that identifying the pericardiacophrenic veins (PCPV), which run parallel to the phrenic nerves [2–4], would aid the differentiation of thymic and non-thymic tumors because the phrenic nerves may be used as a landmark to locate the border of the thymus [5].

The purpose of this study was: 1) to evaluate PCPV depictability as landmarks to locate the phrenic nerves on multi-detector-row CT (MDCT) with or without ECG gating; and 2) to investigate the usefulness of identifying the PCPV to aid the differential diagnosis of anterior mediastinal lesions.

## Methods

### Subjects

Consecutive 56 patients (34 males and 22 females; age range: 15–80 years, median age: 53 years) with a pathologically confirmed anterior mediastinal lesion seen between 2004 and 2011 were included in this study. All of the CT images were evaluable, so no patients were excluded. Fifty lesions originated from the thymus (33 thymomas, 11 thymic carcinomas, 3 germ cell tumors, 2 malignant lymphomas, and 1 thymolipoma), and 6 lesions were of non-thymic origin (2 sarcomas, a metastatic tumor from renal cell carcinoma, a tuberculoma, Castleman’s disease, and an aneurysm). We used Felson’s classification to define the mediastinal compartment of the lesions. The institutional review board approved this retrospective study and no individual patient consent was required.

### CT acquisition

Contrast-enhanced CT of the chest was performed in all cases before surgical resection (47 cases) or image-guided percutaneous biopsy (9 cases). Twenty-four patients were scanned with a 16-row MDCT scanner (IDTI6; Philips Medical Systems, Cleveland, OH), and 32 were scanned with a 64-slice dual source (DS) CT scanner (Somatom Definition, Siemens Medical Systems, Erlangen, Germany). Our institution introduced the DSCT in 2006, and thereafter, the majority of data for this study were obtained with the DSCT. In all cases, contrast-enhanced CT scans were obtained in the craniocaudal direction during inspiratory breath holding. During the scans, 100 mL of contrast medium (300 mgI/mL) were injected into the antecubital vein at a rate of 2 mL/s using a power injector. The CT data were acquired after a scan delay of 100 s. Of the 56 patients, 22 were scanned using the ECG-gating method and the 64-slice DSCT scanner. The CT scan parameters were as follows: for the non-ECG-gated 16-row MDCT (*n* = 24): mAs, 200; tube voltage, 120 kVp; collimation, 16 × 0.75 mm; rotation time, 0.5 s/rotation; and pitch, 0.9; for the non-ECG-gated 64-slice DSCT (*n* = 10): single source mode; reference mAs, 215; tube voltage, 120 kVp; collimation, 64 × 0.6 mm; rotation time, 0.33 s/rotation; and pitch, 1; and for the ECG-gated 64-slice DSCT (*n* = 22): dual source mode; reference mAs, 320/rotation; tube voltage, 120 kVp; collimation, 64 × 0.6 mm; rotation time, 0.33 s/rotation; and pitch, variable depending on the patient’s heart rate. A retrospective ECG-gating method was used for the ECG-gated CT, and the cardiac phase that exhibited the fewest motion artifacts was selected for the mediastinal tumor evaluations. We did not use ECG gating when the anterior mediastinal lesions did not seem to clearly invade the adjacent organs. Axial CT images were reconstructed using gapless 3-mm-thick slices and a smooth kernel for soft tissue, and were displayed at a window width of 300 Hounsfield Unit (HU) and a level of 30 HU for the mediastinum and a window width of 1500 HU and a level of −550 H.U for the lung. The Volume CT Dose Index (CTDIvol) was recorded in 32 of the 56 cases. Mean ± standard deviation of CTDIvol of CT examination with and without ECG gating was 53.5 ± 16.6 and 12.8 ± 3.08, respectively.

### Image interpretation

Two chest radiologists (observers 1 and 2 with 28 and 10 years of experience, respectively), blinded to the patients’ histopathological diagnoses, assessed the CT scans independently. We divided the mediastinum into 4 sections; i.e., into the cranial and caudal parts on each side of the superior margin of each auricle. We named the right upper part section 1, the right lower part section 2, the left upper part section 3, and the left lower part section 4.

**Fig. 1:**
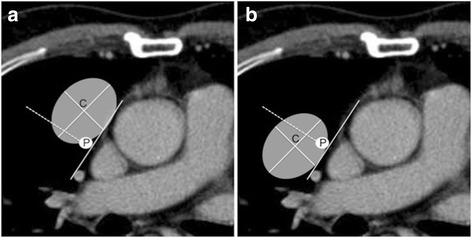
The positional relationships between the center of each anterior mediastinal lesion and the ipsilateral PCPV. We evaluated whether the center of the lesion (C) was located on the medial or lateral side of the ipsilateral PCPV (P). The borderline (dotted line) between the medial and lateral sides was determined as a line vertical to the mediastinal plane. **a** Schema of the center of the lesion located on the medial side of the ipsilateral PCPV. **b** Schema of the center of the lesion located on the lateral side of the ipsilateral PCPV

The PCPV depiction rate in each of the four sections and the spatial relationship between the center of each anterior mediastinal lesion on the trans-axial image where the lesion was the largest and the ipsilateral PCPV, were assessed on each scan. The center of the mediastinal lesion was determined as a point at which the longest axis and the maximum minor axis vertical to the longest axis crossed. The borderline between the medial and lateral sides was determined as a line from the PCPV, which ran vertical to the mediastinal plane (Fig. 1). The PCPV depiction rate was visually scored using a 4-point scale: detectable in <25% of slices: 1; 25–50%: 2; 50–75%: 3; and 75–100%: 4. The differences in the PCPV depiction rate between ECG-gated and non-ECG-gated CT were assessed statistically. The two radiologists learned the anatomy and typical CT images of the PCPV beforehand. Any difference in interpretation was solved by consensus of the two observers. When PCPV depiction was poor around the anterior mediastinal mass, we determined the relationship between the locations of the lesion and PCPV, to complement the PCPV, by the detectable parts of the PCPV located at the cranial and caudal portions of the invisible area.

### Evaluation of phrenic nerve resection and palsy

Frequency of phrenic nerve conservation and resection at surgery and occurrence of phrenic nerve palsy after surgery were evaluated from the medical record.

### Statistical analysis

We used the Wilcoxon rank sum test for comparisons of the PCPV depiction rates obtained for each section with and without ECG-gating. The Wilcoxon signed-ranked test was used to compare the PCPV depiction rates for the left and right sides. These two tests were carried out using a software MEPHAS on the web (http://www.gen-info.osaka-u.ac.jp/MEPHAS/). Fisher’s exact test was used to assess the relationships between the location of each anterior mediastinal lesion relative to the PCPV and the histological diagnosis, with a software js-STAR (http://www.kisnet.or.jp/nappa/software/star/). *P*-values of less than 0.05 were considered to indicate significant differences.

## Results

### PCPV depiction rate for each region

**Fig. 2:**
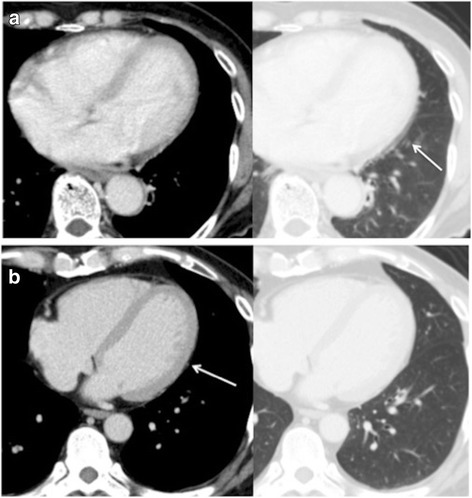
**a** Axial non-ECG-gated CT scan obtained in a case with a PCPV depiction rate score of 1. The PCPV was not detectable in the left lower part of the mediastinum because of cardiac motion artifacts (arrow). **b** Axial ECG-gated CT scan obtained in a case with a PCPV depiction rate score of 4. The PCPV was clearly depicted in the left lower part of the mediastinum (arrow)

The PCPV depiction rates for each mediastinal section obtained by each observer are shown in Table 1. When a depiction rate score of 3 or more on both the right and left sides was defined as indicating overall good depiction of the PCPV, 20/56 (36%) on the right side and 28/56 (50%) on the left side were judged as good depiction by observer 1, and 17/56 (30%) and 28/56 (50%), respectively, by observer 2. The differences in each PCPV depiction rate between non-ECG-gated and ECG-gated CT are shown in Table 2. Regarding the PCPV depiction rates obtained with or without ECG gating by observer 1, the PCPV depiction rate for the lower left section of the mediastinum was significantly higher when ECG gating was used (*p* = 0.0032) (Fig. 2). During the evaluations performed by observer 2, the PCPV depiction rate for the lower left section of the mediastinum tended to increase during ECG gating (*p* = 0.069).

**Table 1:** PCPV depiction rates for each part of the mediastinum

	PCPV depiction rate score			
Observer 1	1	2	3	4
Section 1	14 (25)	15 (27)	16 (29)	11 (20)
Section 2	8 (14)	13 (23)	17 (30)	18 (32)
Section 3	4 (7)	8 (14)	15 (27)	29 (52)
Section 4	10 (18)	14 (25)	17 (30)	15 (27)
Observer 2				
Section 1	17 (30)	17 (30)	10 (18)	12 (21)
Section 2	10 (18)	10 (18)	25 (45)	11 (20)
Section 3	6 (11)	8 (14)	14 (25)	28 (50)
Section 4	14 (25)	9 (16)	18 (32)	15 (27)

The figures in parentheses are percentages

*PCPV* pericardiacophrenic veins

**Table 2:** Differences in the PCPV depiction rates for each part of the mediastinum between non-ECG gated and ECG gated CT

	PCPV depiction rate score				
Observer 1	1	2	3	4	*p*-value
Section 1					
Non-ECG gated	11 (32)	10 (29)	6 (18)	7 (21)	0.14
ECG gated	3 (14)	5 (23)	10 (45)	4 (18)	
Section 2					
Non-ECG gated	5 (15)	9 (26)	11 (32)	9 (26)	0.35
ECG gated	3 (14)	4 (18)	6 (27)	9 (41)	
Section 3					
Non-ECG gated	3 (9)	5 (15)	8 (24)	18 (53)	0.96
ECG gated	1 (5)	3 (14)	7 (32)	11 (50)	
Section 4*					
Non-ECG gated	9 (26)	10 (29)	10 (29)	5 (15)	0.0032
ECG gated	1 (5)	4 (18)	7 (32)	10 (45)	
Observer 2					
Section 1					
Non-ECG gated	11 (32)	12 (35)	3 (9)	8 (24)	0.54
ECG gated	6 (27)	5 (23)	7 (32)	4 (18)	
Section 2					
Non-ECG gated	7 (21)	7 (21)	14 (41)	6 (18)	0.34
ECG gated	3 (14)	3 (14)	11 (50)	5 (23)	
Section 3					
Non-ECG gated	3 (9)	6 (18)	9 (26)	16 (47)	0.69
ECG gated	3 (14)	2 (9)	5 (23)	12 (55)	
Section 4					
Non-ECG gated	11 (32)	6 (18)	10 (29)	7 (21)	0.069
ECG gated	3 (14)	3 (14)	8 (36)	8 (36)	

*ECG* electrocardiographic, *PCPV* pericardiacophrenic veins

* The differences between the data obtained using non-ECG gated and ECG gated CT were statistically significant (*p* < 0.05). The figures in parentheses are percentages

### Relationships between the location of each anterior mediastinal lesion relative to the PCPV and the histological diagnosis

**Fig. 3:**
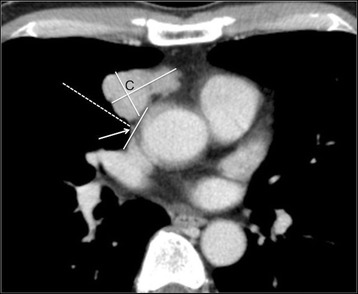
Axial CT detected a thymoma in the anterior mediastinum. The center of the tumor (C) was located on the medial side of the ipsilateral PCPV (arrow)

**Fig. 4:**
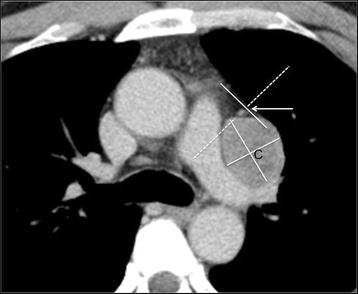
A case involving a metastatic tumor derived from renal cell carcinoma. The center of the lesion (C) was located on the lateral side of the ipsilateral PCPV (arrow)

All 50 thymic tumors and 3 of the 6 non-thymic lesions were located on the medial side of the ipsilateral PCPV (Fig. 3). The 3 lesions located on the lateral side of the ipsilateral PCPV were confirmed to be non-thymic (Castleman’s disease, a metastatic tumor from renal cell carcinoma (Fig. 4), and a tuberculoma) (*p* = 0.0007) (Table 3).

**Table 3:** Relationships between the location of the center of each anterior mediastinal lesion relative to the ipsilateral PCPV and lesion type

	Medial	Lateral	Total
Thymic origin	50	0	50
Non-thymic origin	3	3	6
	53	3	56

*PCPV* pericardiacophrenic veins

### Phrenic nerve resection and palsy

Nine of the 56 (16%) cases underwent phrenic nerve resection due to tumor invasion to the nerve, which resulted in postoperative phrenic nerve palsy in all cases. On the other hand, the phrenic nerves could be preserved in 4 of the 56 (7%) cases despite that they were surrounded by the tumors. In one of the 4 cases, mild phrenic nerve palsy developed even after preservation of the nerve at surgery.

## Discussion

Our results indicate the depictability of the PCPV, which runs parallel to the phrenic nerves, on MDCT. Relatively good depiction of the PCPV was obtained in the left upper part of the mediastinum. The PCPV depiction rate in the left lower part of the mediastinum was improved significantly by the ECG-gating technique. All 3 lesions located on the lateral side of the ipsilateral PCPV were diagnosed as non-thymic tumors based on the spatial relationship between their centers and the ipsilateral PCPV. These results indicate that the locations of the bilateral phrenic nerves can be determined by identifying the PCPV on MDCT. ECG-gated CT may reduce cardiac motion artifacts in the left lower part of the heart, resulting in better depiction of the PCPV. When the center of an anterior mediastinal lesion is located on the lateral side of the PCPV, non-thymic lesions should be considered during diagnosis.

**Fig. 5:**
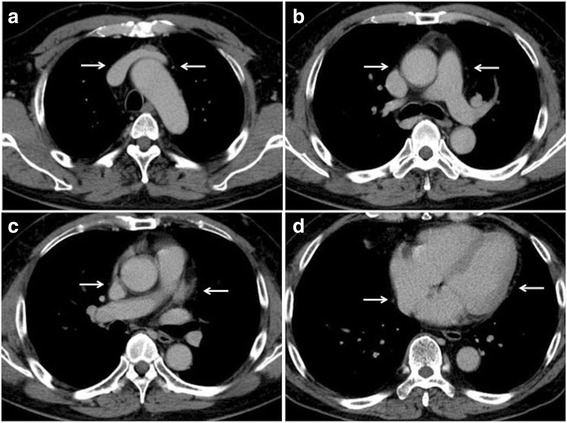
Representative location of the bilateral PCPV, which runs parallel to the phrenic nerves The PCPV on each CT slice is shown as dotted structures (arrows). **a** On the right side, the phrenic nerve lies laterally to the right brachiocephalic vein and the superior vena cava, while the left phrenic nerve runs laterally to the aortic arch. **b**-**d** The bilateral phrenic nerves pass anteriorly to the respective pulmonary hila and inferiorly in a broad vertical plane along the margin of the heart between the parietal pericardium and the mediastinal pleura.

The phrenic nerves innervate the diaphragm to provide motor functions and the central intrathoracic and peritoneal surfaces of the diaphragm to provide sensory functions [6]. The phrenic nerves originate from the cervical C3-C5 nerves and run from the thoracic inlet to the diaphragm along the lateral part of the mediastinum [6, 7]. The right phrenic nerve lies laterally to the right brachiocephalic vein and the superior vena cava, while the left phrenic nerve runs laterally to the aortic arch. Both phrenic nerves then pass anteriorly to their respective pulmonary hila and inferiorly in a broad vertical plane along the margin of the heart between the fibrous (parietal) pericardium and the mediastinal pleura (Fig. 5) [6]. The PCPV, which have connections to the pericardium, pleura, and diaphragm, ascend towards the phrenic nerves between the parietal pericardium and mediastinal pleura. The PCPV are sometimes connected superiorly to the internal thoracic, left superior intercostal, and brachiocephalic veins. The left PCPV feeds into the left brachiocephalic vein via an opening opposite the orifice for the left jugular vein, while the right PCPV feeds into the ipsilateral brachiocephalic vein at a more proximal site. In cases involving the occlusion of the superior vena cava and azygos vein, the PCPV can increase in diameter due to the development of collateral blood vessels [2–4]. This anatomical information regarding the PCPV can help clinicians to identify the location of the phrenic nerves.

There have been a few papers about the depiction of the PCPV or the phrenic nerves on CT [3, 4, 6–10], but no previous studies have examined the relationship between the locations of the phrenic nerves and anterior mediastinal lesions. It is difficult to visualize the phrenic nerves on CT, as they are encased in folds of the parietal pleura and mediastinal fat, but the PCPV can be used as landmarks to locate the phrenic nerves [8]. The pericardiacophrenic artery (PCPA) also runs parallel to the phrenic nerve, but the PCPA is usually smaller than the PCPV in diameter, and we used images at the delayed contrast enhancement phase. So, we evaluated the PCPV rather than the PCPA. In a previous study, the phrenic nerve depiction rate on CT images with a slice thickness of 8 mm was 85% (11/13) on the left side, 8% (1/13) on the right side, and 8% (1/13) for the bilateral nerves [9]. A study based on coronary 64-row MDCT indicated that the phrenic nerves were detected in 78 of 106 cases (74%) on the left side and 50 cases (47%) on the right side [10]. The PCPV depiction rate on the left side was also higher in our study than on the right side. It might be due to the location of the upper part of the right phrenic nerve that runs closely to the right brachiocephalic vein and superior vena cava. It would be difficult to separate these veins and the right phrenic nerve. On the other hand, the upper part of the left phrenic nerve tends to separate from the subaortic arch, leading to the difference in detectability between the right and left phrenic nerves. The phrenic nerve is a tiny structure with a diameter of 1–3 mm [9]. So, high spatial resolution of CT could help detect the nerve. We did not employ thin slice images, and evaluated 3-mm-thick images for routine clinical use. The ECG gating technique might be useful for lesions located at the left lower part (Section 4), since the region is usually most affected by cardiac motion. In this section, the PCPV depiction rate improved by ECG gating in observer 1 (*p* = 0.0032) and tended to improve in observer 2 (*p* = 0.069). The difference between the two observers may be due to the differences in their experience with chest radiology and the ECG-gating technique. The ECG-gating technique is rather unusual as a preoperative examination. So, we think that the ECG-gating technique might be useful for patients with an anterior mediastinal lesion, especially adjacent to the left ventricle of the heart, and for whom evaluation of anatomical relationship between the phrenic nerves and mediastinal lesion is considered clinically important.

Thymoma, thymic carcinoma, thymic cyst, mature teratoma, malignant germ cell tumor, and malignant lymphoma are representative anterior mediastinal tumors [11, 12]. However, other lesions such as metastases and tuberculosis infections also occur in the anterior mediastinum [12]. Identifying the phrenic nerves and evaluating the positional relationships between anterior mediastinal lesions and the phrenic nerves may aid the differentiation of anterior mediastinal lesions; i.e., it could help to determine whether a lesion is of thymic origin. The 3 lesions located on the lateral side of the ipsilateral PCPV were of non-thymic origin in this study. Of these lesions, the treatment strategy for the metastasis from renal cell carcinoma and tuberculoma might have been changed to a non-surgical one if these lesions had been diagnosed preoperatively.

It is clinically important to detect the phrenic nerves during preoperative CT evaluations. Combined resection of the phrenic nerves and mediastinal tumors could impair respiratory function and cause severe postoperative complications. Preoperative evaluations to determine whether a tumor has invaded the phrenic nerves are clinically important because patients with thymomas that have invaded the phrenic nerves may be candidates for neoadjuvant chemotherapy in order to prevent phrenic nerve resection. Conservation of the phrenic nerves could result in better postoperative respiratory function and prognosis. In the present study, the phrenic nerve could be preserved in 4 patients despite the tumors surrounding the nerve, and only one of them developed mild phrenic nerve palsy. Thus, clinical outcome of the patients could be improved by preoperatively evaluating the relationship between the phrenic nerve and mediastinal tumor and determining the strategy for manupulation of the phrenic nerve. In addition, during extended thymectomy for thymoma combined with myasthenia gravis the lateral resection border is basically defined by the phrenic nerves. Therefore, the detection of the phrenic nerves on CT is useful to determine the surgical field preoperatively [5]. It may also be useful to detect extensions of the phrenic nerves in order to diagnose neurogenic tumors derived from the phrenic nerves and identify the cause of cases of phrenic nerve paralysis.

The main limitation of this study was that it did not include many non-thymic tumors. However, we assume that the information regarding the positional relationships between the phrenic nerves and anterior mediastinal tumors obtained in this study may aid decisions as to whether anterior mediastinal tumors are thymic in origin. So, further investigations of such tumors are desirable. In addition, we did not evaluate relationship between the amount of mediastinal fat and PCPV depiction; the depiction rate might become higher if the mediastinal fat is rich, because the PCPV would separate from the heart.

## Conclusions

We evaluated the PCPV depiction rate, and it was suggested that the use of the ECG-gating technique during MDCT improves the depictability of the PCPV in the left lower section of the anterior mediastinum. Solitary anterior mediastinal lesions located on the lateral side of the ipsilateral PCPV are likely to be non-thymic.

## Abbreviations

CT: computed tomography
DS: dual source
ECG: electrocardiographic
MDCT: multi-detector-row computed tomography
PCPV: pericardiacophrenic veins
